# A Novel Flow Cytometric High Throughput Assay for a Systematic Study on Molecular Mechanisms Underlying T Cell Receptor-Mediated Integrin Activation

**DOI:** 10.1371/journal.pone.0006044

**Published:** 2009-06-25

**Authors:** Kwangmi Kim, Lin Wang, Inkyu Hwang

**Affiliations:** Department of Chemistry and Chemical Biology, The Scripps Research Institute, La Jolla, California, United States of America; Karolinska Institutet, Sweden

## Abstract

Lymphocyte function-associated antigen 1 (LFA-1), a member of β2-integrin family, exerts multiple roles in host T cell immunity and has been identified as a useful drug-development target for inflammatory and autoimmune diseases. Applying the findings that primary resting T cells absorb nanometric membrane vesicles derived from antigen presenting cells (APC) via dual receptor/ligand interactions of T cell receptor (TCR) with cognate peptide-major histocompatibility complex (MHC) complex (pMHC) and LFA-1 with its ligand, intercellular adhesion molecule-1 (ICAM-1), and that signaling cascades triggered by TCR/pMHC interaction take a part in the vesicle-absorption, we established a cell-based high throughput assay for systematic investigation, via isolation of small molecules modulating the level of vesicle-absorption, of molecular mechanisms underlying the T cell absorption of APC-derived vesicles, i.e., structural basis of TCR/pMHC and LFA-1/ICAM-1 interactions and TCR-mediated LFA-1 activation. As primary T cells along with physiological ligands expressed in biological membrane are used and also individual cells in assay samples are analyzed by flow cytometry, results obtained using the assay system hold superior physiological and therapeutic relevance as well as statistical precision.

## Introduction

Lymphocyte function-associated antigen 1 (LFA-1), an integrin composed of αL (CD11a) and β_2_ (CD18) subunits, plays multiple roles in T cell immunity [1]. It plays a pivotal role in T cell activation by generating costimulatory signals and promoting formation of immunological synapse (IS) at the junction of T cell-antigen presenting cell (APC) contact. It also plays an important role in migration of effector T cells to the sites of infection and inflammation by assisting transmigration of those T cells across the vasculature. It is also involved in execution of effector T cell functions by directing guided delivery of effector molecules to specific target cells [2]. In addition, LFA-1 is implicated in various immunologic diseases such as chronic inflammation and autoimmune diseases and has been identified as a target for development of drugs against those diseases [3], [4].

As with other integrins, the functional activity of LFA-1 is controlled by a form of intracellular signaling, termed ‘inside-out’ signaling [5], [6]. When the ‘inside-out’ signal is induced by interaction of specific receptors with their ligands, e.g., interaction of T cell receptor (TCR) with cognate peptide/MHC complexes (pMHCs), both affinity and avidity of LFA-1 for its ligand, intercellular adhesion molecule-1 (ICAM-1), are enhanced by its conformational change and spatial rearrangement.

Exosomes are nanometric membrane vesicles formed inside cells and released into the extracellular space [7]. Previously, we reported that purified CD8^+^ 2C TCR transgenic (Tg) T cells absorbed exosome-like membrane vesicles (eMVs) purified from culture supernatants of artificial APCs, namely Drosophila (Dros) cells expressing various mouse immuno-molecules required for APC function, and cultured dendritic cell (DC) line [8], [9], [10]. The eMV-absorption by 2C T cells was found to be mediated by specific dual receptor/ligand interactions of 2C TCR with a cognate pMHC (L^d^/QL9 or L^d^/p2Ca) plus LFA-1 with ICAM-1. Further, in this study, we reveal that the vesicle-absorption also involves intracellular signaling cascades initiated by TCR triggering and required for LFA-1 activation.

Chemical genetics, a research paradigm for using small chemical compounds to modify protein function(s) to alter phenotype(s) of cells or entire organisms, is emerging as a powerful tool not only for uncovering molecular mechanisms underlying important biological phenomena but also for exploring new therapeutics [11], [12]. High-throughput screening (HTS) is a key element of chemical genetic approaches and thus developing HTS platforms with an innovative concept, simplicity and statistical precision is pivotal for successful chemical genetic studies. Here, we introduce a novel high throughput assay for a systematic investigation of molecular mechanisms controlling TCR-mediated LFA-1 activation. Given that ex vivo purified primary T cells along with physiological ligands expressed in biological membranes are used and that individual cells in assay samples are interrogated by flow cytometry, this HTS system features superior physiological and therapeutic relevance, high sensitivity and statistical precision.

## Results

### Preparation of plasma membrane-derived microvesicles

The amount of eMVs collected from culture supernatants is limited. To improve the yield of microvesicles expressing immuno-molecules of interest, we prepared vesicles from the purified plasma membrane fraction of Dros APCs.

EM pictures showed that sizes of plasma membrane-derived vesicles (pMVs) prepared from Dros APCs expressing L^d^ plus B7-1 and ICAM-1 (L^d^B7-1ICAM-1 Dros APCs) were relatively uniform (approximately 200–300 nm in diameter) (Supplementary Figure S1A). Accordingly, we were able to estimate the average number of each mouse immuno-molecule expressed in the pMVs (Supplementary Figure S1B and C).

**Figure 1:**
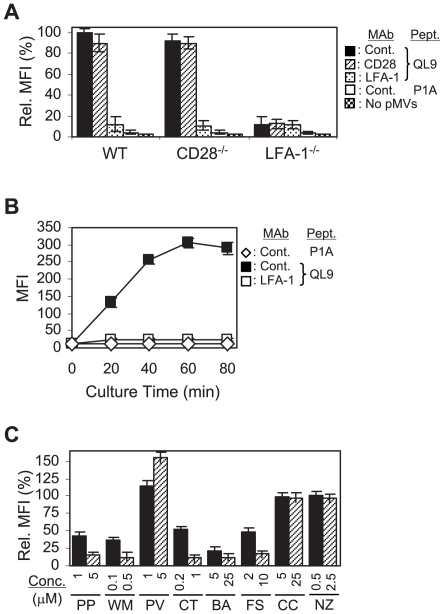
Importance of intracellular signaling cascades in the pMV-absorption. (A) Wild type (WT), CD28^−/−^, and LFA-1^−/−^ 2C T cells, pre- treated with the respective mAbs, were cultured with or without the peptide-loaded L^d^B7-1ICAM-1 pMVs, as indicated, followed by mAb staining for B7-1 and flow cytometric analysis. Mean fluorescence intensities (MFIs) of B7-1 staining relative to that of WT 2C T cells, pre-treated with control mAb and cultured with the QL9-loaded pMVs, were plotted. Note that P1A peptide forms complex with L^d^ but L^d^/P1A complex is not recognized by 2C TCR [8]. (B) WT 2C T cells, pre-treated with the respective mAbs, were cultured with the peptide-loaded pMVs for different periods of time, as indicated, and stained for B7-1. (C) WT 2C T cells were treated with PP2 (PP), Wortmannin (WM), Pervanadate (PV), Cytochalasin D (CT), BAPTA/AM (BA), Forskolin (FS), Cyclosporin A (CC) and Nocodazole (NZ), respectively, as indicated, before culture with the QL9-loaded pMVs. MFIs of the B7-1 staining relative to that of 2C T cells treated with DMSO alone were plotted.

We used pMVs expressing L^d^ plus B7-1 and ICAM-1 (L^d^B7-1ICAM-1 pMVs) for the following reasons. Firstly, lack of B7-1 expression in resting T cells and high level expression of B7-1 in the pMVs allow us to employ B7-1 mAb staining of T cells as a readout to measure the extent of pMV-absorption by T cells with low background and high sensitivity (Figure 1A and Supplementary Figure S2A). Secondly, using 2C T cells deficient in CD28 or LFA-1 expression and mAbs blocking interactions of those receptors with their ligands, it is feasible to probe the contributing roles of TCR/pMHC, CD28/B7-1 and LFA-1/ICAM-1 interactions in the pMV-absorption.

### 2C T cell absorption of L^d^B7-1ICAM-1 Dros pMVs

As for absorption of Dros eMVs [8], 2C T cells absorbed Dros pMVs only when the latter were loaded with an agonistic peptide (QL9 or p2Ca) [13] (Figure 1A and Supplementary Figure S2A).

LFA-1/ICAM-1 interaction played a critical role in the pMV-absorption. Thus, wild type and CD28^−/−^ 2C T cells treated with anti-LFA-1 (CD11a) mAb and 2C.LFA-1^−/−^ T cells absorbed the pMVs at a greatly reduced level (Figure 1A and B). In contrast, CD28/B7-1 interaction appeared to play only a marginal role in the absorption. The level of pMV absorption increased progressively as the culture time extended (Figure 1B).

We examined 2C T cells cultured with the pMVs with confocal microscopy (Supplementary Figure S2B). The absorbed pMVs visualized as punctate B7-1 staining were easily found on the cell surface when 2C T cells were cultured with the QL9 (or P2Ca)-loaded pMVs.

### Involvement of intracellular signaling in the pMV-absorption

As LFA-1 expressed in resting naïve T cells exists mainly in inactive low affinity state [6], results that LFA-1 played a key role in the pMV-absorption suggested the transition of LFA-1 to active high affinity state in the course of the pMV-absorption, which could occur via ‘inside-out’ signaling induced by engagement of 2C TCRs with L^d^/QL9 (or L^d^/P2Ca) complexes [14].

We investigated the possible involvement of intracellular signaling in the pMV-absorption by treating 2C T cells with various pharmacological agents targeting specific signaling molecules (Figure 1C).

Treatment of 2C T cells with PP2 and Wortmannin, inhibitors of protein tyrosine kinases (PTKs) and phosphoinositide 3-kinase (PI3-K), respectively, dramatically reduced the level of pMV-absorption, while treatment with Pervanadate, a protein tyrosine phosphatase inhibitor, significantly elevated the level of pMV-absorption.

Treatment with Cytochalasin D (an inhibitor of actin polymerization), BAPTA/AM (a membrane permeable intracellular Ca^2+^ chelator) and Forskolin (an activator of adenylyl cyclase) also inhibited the pMV-absorption.

Treatment of 2C T cells with Cyclosporin A (an inhibitor of Ca^2+^-dependent serine/threonine phosphatase) and Nocodazole (a tubulin antagonist) had little effect on the pMV absorption. Together, these results revealed the importance of specific intracellular signaling cascades in the pMV-absorption.

### Induction of F-actin polymerization during culture of 2C T cells with the pMVs

It is well known that TCR triggering promptly induces F-actin polymerization via activation of signaling pathways involving PTKs, PI3-K, Vav, Rac and Abi/Wave complex which are also known to play a role in integrin activation [15], [16], [17].

**Figure 2:**
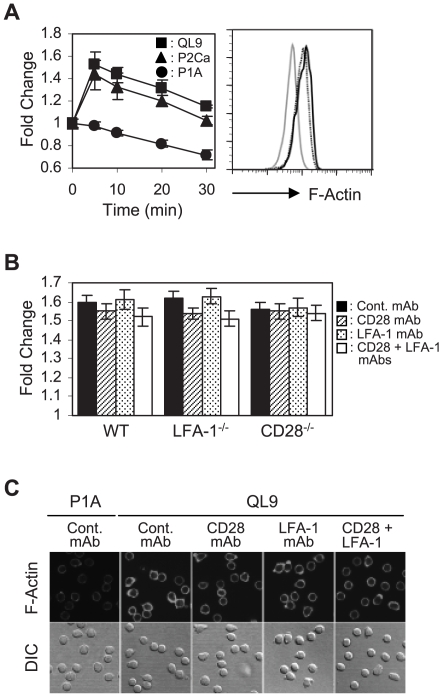
Analysis of F-actin polymerization occurring during culture of 2C T cells with the pMVs. (A) Wild type (WT) 2C T cells were cultured with the peptide-loaded pMVs for different periods of time and stained for F-actin with FITC-labeled phalloidin followed by flow cytometric analysis. Fold changes in the level of F-actin relative to that of 2C T cells fixed immediately after mixing with the QL9-loaded L^d^B7-1ICAM-1 pMVs were plotted. Histograms represent the F-actin staining of 2C T cells cultured with P1A- (solid grey), P2Ca- (dotted black) and QL9- (solid black) loaded pMVs for 5 min, respectively. (B) WT, CD28^−/−^ and LFA-1^−/−^ 2C T cells were treated with the respective mAbs, as indicated, before 5 min culture with the QL9-loaded pMVs. Fold changes in the level of F-actin during the culture were plotted. (C) WT 2C T cells were pre-treated with the respective mAbs and cultured with the peptide-loaded pMVs, as indicated, for 20 min on poly-L-lysine-coated coverslips followed by staining for F-actin with FITC-labeled phalloidin.

Flow cytometric analysis showed that F-actin contents of 2C T cells rapidly increased following culture with the pMVs loaded with either QL9 or p2Ca peptide. The level of F-actin in those cells reached a peak within 5 minutes of the culture and remained elevated for at least 30 minutes (Figure 2A).

Treatment of wild-type 2C T cells with anti-LFA-1 and anti-CD28 mAbs had little effect on the F-actin polymerization (Figure 2B). In line, 2C.LFA-1^−/−^ and 2C.CD28^−/−^ T cells cultured with the QL9-loaded pMVs showed comparable levels of F-actin content as wild-type 2C T cells.

Consistent with results from the flow cytometric analysis, microscopic analysis showed more prominent cortical F-actin staining in 2C T cells cultured with the pMVs loaded with QL9 peptide than in control T cells and both anti-LFA-1 and anti-CD28 mAb blocking exerted little effect on the level of cortical F-actin (Figure 2C).

Reflecting critical roles of PTKs and PI3-K in early membrane-proximal TCR signaling [18], PP2 and Wortmannin treatments strongly inhibited the F-actin polymerization (Supplementary Figure S3A). In addition, activation of PTKs and PI3-K, exemplified by phosphorylation of phospholipase C-γ1 (PLC-γ1) and Akt, respectively, were readily detected shortly after culture with the QL9-loaded pMVs (Supplementary Figure S3B and C).

**Figure 3:**
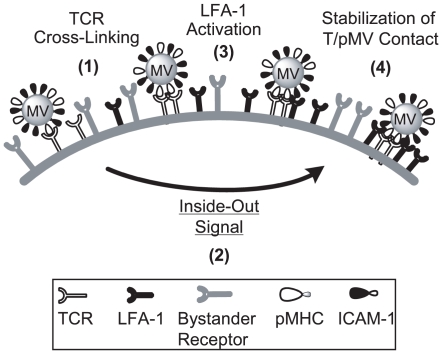
Schematic representation of the mechanistic model for 2C T cell absorption of the pMVs. The whole process of the pMV-absorption commences as 2C TCRs interact with cognate pMHCs expressed on the pMVs. The TCR/pMHC interaction activates intracellular signaling cascades (i.e., “inside-out” signaling) resulting in promotion of LFA-1 function and thereby stabilization of T/pMV contact. According to that model, the pMV-absorption can be inhibited by three different routes: (i) interruption of TCR/pMHC interaction, (ii) interruption of LFA-1/ICAM-1 interaction, (iii) inhibition of intracellular signaling cascades for LFA-1 activation.

Together, these results illustrated that the sole interaction of 2C TCR with cognate pMHC expressed in L^d^B7-1ICAM-1 pMVs could trigger signaling events involved in LFA-1 activation process (Figure 3).

### High throughput screening for systematic study on molecular mechanisms underlying the pMV-absorption

**Figure 4:**
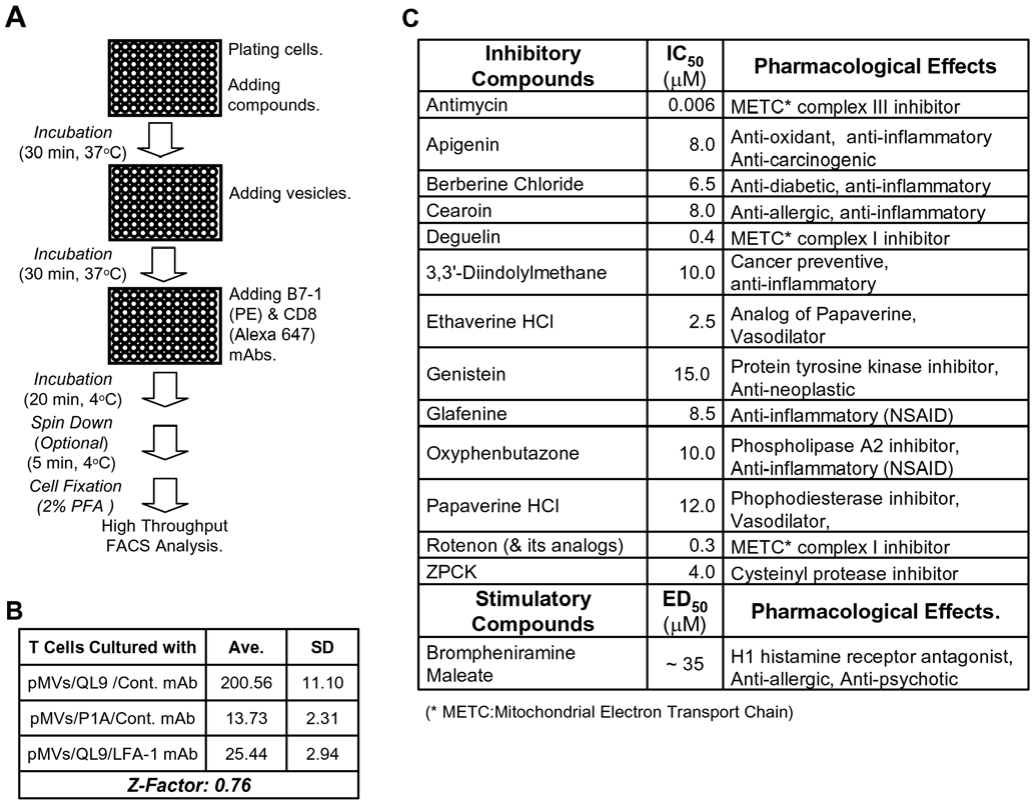
High throughput screening for systematic study on molecular mechanisms underlying the pMV-absorption. (A) Flow diagram of the high throughput screening (HTS) for isolation of small molecules modulating the pMV-absorption. (B) Z-factor was calculated with averages and standard deviations of 32 sets of assays; each set included assays with control mAb plus QL9-loaded pMVs used as the positive control, anti-LFA-1 mAb plus QL9-loaded pMVs used as the negative control and with control mAb plus P1A-loaded pMVs. (C) IC_50_s (or ED_50_) and pharmacological uses of hits isolated from the pilot scale HTS were summarized.

The assay system for detecting the pMV-absorption has several features suitable for the systematic investigation of molecular mechanisms governing TCR-mediated LFA-1 activation. Firstly, while the pMV-absorption proceeds via complex cellular and molecular mechanisms (Figure 3), the assay itself is simple and expeditious and thus can be easily implemented as a high throughput assay (Figure 4A). Secondly, as primary T cells along with physiological ligands expressed in biological membranes are employed, results obtained from the experiments may have superior physiological relevance. Thirdly, as flow cytometry, which enables interrogation of individual cells with multiple parameters, is employed in the assay, only a selected population of cells in the samples, e.g., CD8^+^ and live T cells, can be included in data analysis and thus interpretation of assay results and identification of false positives are facilitated. In addition, because repeated washes, which are time-consuming and likely generate experimental errors but are often imperative in other types of assays using a fluorescence-tagged material for reducing the level of background, are not necessary, reliability of the assay becomes high; due to fluidic operation of flow cytometry that takes and carries cells with a stream of sheath buffer to the laser interrogation point, residual free fluorescent reagents in the samples does not hamper analysis of samples. Indeed, Z-factor of the assay, a parameter widely used as a measure of variability and reproducibility (i.e., intra-assay variation) of high throughput assay, turned out highly desirable (Figure 4B). Inter-assay variation, inferred by average and standard deviation (SD) of Z-factors drawn from four separate sets of experiments, was also found favorable; the average and SD were 0.72 and 0.046, respectively.

In order to evaluate the practicality and effectiveness of the high throughput assay, we attempted to screen a 2000-membered chemical library (The Spectrum Collection™, MicroSource Discovery, Inc.) composed of both natural and synthetic small molecules whose biological functions have been identified to different extents. We screened the library using 96 well format in conjunction with an automatic sampler (BD High Throughput Sampler, BD Bioscience) equipped to a flow cytometer (BD FACSCalibur); compounds were initially tested at 40 µM. After completion of the HTS campaign, compounds showing over 75% inhibition of the pMV-absorption were selected and retested twice in duplicate to confirm the activity.

In summary of the HTS campaign, total 53 hits were isolated in the primary screening and 46 compounds showed the reproducible activity; a false positive rate and a reconfirmation rate of the HTS were approximately 0.4% (7 compounds out of 2000 compounds) and 86% (46 compounds out of 53 compounds), respectively (http://www.mbcfoundation.org/pdfs/Lipinski20Keynote20address.pdf).

Potencies (IC_50_) and toxicities of hits showing the reproducible activity were examined testing them at titrated concentrations; to this end, instead of being fixed in paraformaldehyde, 2C T cells were resuspended in a propidium iodide (PI)-containing buffer at the end of the assay and immediately analyzed by flow cytometry. Compounds exhibiting a high potency (IC_50_<15 µM) and no cytotoxicity (based on PI exclusion) at IC_50_ were isolated as final hits.

Here, a few hits deserve to be highlighted (Figure 4C). Inhibition of the pMV-absorption by Genistein [19], a protein tyrosine kinase inhibitor, appears consistent with the inhibition by PP2 (Figure 1C). Papaverine and Ethaverine share the same core chemical structure (Supplementary Figure S4A) and are used as vasodialators. Papaverine has an inhibitory activity for phosphodiesterase; thus, Papaverine treatment may increase the cellular concentration of cAMP [20]. Given that, inhibition of the pMV absorption by Papaverine appears in line with the inhibition by Forskolin which activates adenylyl cyclase resulting in increase in concentration of cAMP [21] (Figure 1C). Inhibitions of the pMV-absorption by Berberine chloride [22], Cearoin [23], 3,3′-Diindoylmethane [24], Glafenine [25] and Oxyphenbutazone [26] are intriguing for their known anti-inflammatory activity. Inhibition by ZPCK, a cysteinyl protease inhibitor [27], is also worth mentioning for the role of protease in LFA-1 activation [28].

### Promotion of the pMV-absorption by structurally-related histamine receptor antagonists and antidepressants

**Figure 5:**
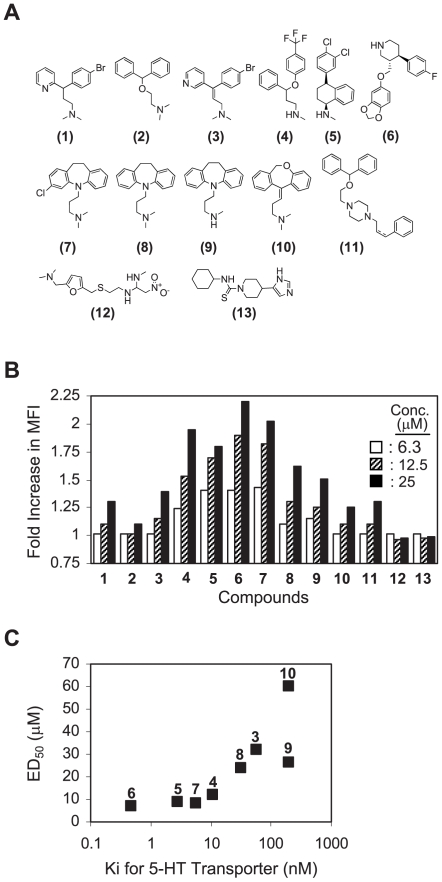
Structure-activity relationship study on the agonistic activity of Brompheniramine on the pMV-absorption. (A) Chemical structures of structurally- or functionally-related compounds of Brompheniramine are shown. Refer to Supplementary Figure 4 for their names and pharmacological uses. (B) 2C T cells were treated with compounds shown in (A) at titrated concentrations and cultured with the QL9-loaded pMVs followed by mAb staining for B7-1. MFIs of the B7-1 staining relative to that of 2C T cells treated with DMSO alone were plotted. Experiments were performed in duplicate and standard deviations of duplicate samples were all within 10% of the averages. Shown is one of three representative experiments. (C) ED_50_s of the compounds were calculated from the experiments described in (B) and plotted against Kis of the compounds for 5-HT transporter. Note: Ki values used in this plot were adopted from PDSP Ki data base (http://pdsp.med.unc.edu/pdsp.php). To ensure data consistency, only the Ki values obtained using rat frontal cortex as the source of 5-HT transporter and [^3^H]-5-HT as the hot ligand were used [45].

Another hit worth noting is Brompheniramine maleate (BPM), a clinically used H1 histamine receptor antagonist [29]. Different from other hits, it was selected due to its agonistic activity on the pMV-absorption. Namely, the BPM treatment significantly augmented the level of pMV absorption by 2C T cells; EC_50_ of BPM was determined at 35 µM. The highly reproducible activity of BPM led us to examine other structurally- or functionally-related molecules (Figure 5A for chemical structures and Supplementary Figure S4B for their names and pharmacological uses). In summary, molecules sharing analogous structural moieties with BPM, i.e., diaryl and alkylated amine functionalities, revealed the common agonistic activity. Notably, among the molecules tested, clinically available antidepressants with stronger serotonin reuptake inhibition activity [30] (i.e., Fluoxetine, Sertraline, Paroxetine, Chlomipramine) showed the superior activity (Figure 5B and C and Supplementary Figure S5).

## Discussion

Encounter of T cells with APC carrying cognate pMHCs leads to formation of TCR microclusters followed by generation of immunological synapse (IS) at the interface of T/APC contact [31]. While the formation of IS is vital for promoting firm and stable T/APC contact and eliciting sustainable intracellular signals, key intracellular signaling events for T cell activation are elicited prior to IS formation, at the stage of TCR microcluster formation. Considering the size of L^d^B7-1ICAM-1 pMV and the number of pMHCs expressed on the pMVs, the process of 2C T cell absorption of the pMVs may mimic the early stage of T-APC interaction leading to and immediately following the formation of TCR microclustering.

Since LFA-1 is expressed in low-affinity/avidity form in resting naïve T cells [6], the finding that LFA-1 played a pivotal role in the pMV absorption by 2C T cells (Figure 1A) was intriguing and suggested the involvement of ‘inside-out’ signaling evoked by TCR stimulation. Involvement of intracellular signaling in pMV-absorption was clearly illustrated by the experiments using pharmacological reagents to inhibit the activity of specific signaling molecules (Figure 1C).

Results that the interaction of 2C TCR with a cognate pMHC on the pMVs was necessary and sufficient for the F-actin polymerization are in agreement with the results of others [16], [17] and showed that the signaling process involved in the pMV-absorption was initiated upon TCR triggering.

In view of the above results, we propose a mechanistic model that 2C T cell absorption of L^d^B7-1ICAM-1 pMV is preceded by the activation of LFA-1 (Figure 3). Prior to LFA-1 activation, the interaction of 2C TCRs with the cognate pMHCs may be too weak and transient to establish stable pMV absorption. Here, strong binding may hinge crucially on prior TCR-dependent ‘inside-out’ signaling to activate LFA-1 function.

Applying the assay for detecting the pMV-absorption, we established a HTS platform. Use of primary cells in study of biological phenomena is considered ideal for gathering physiologically relevant results. Nevertheless, difficulty of securing large number of primary cells often makes it impractical to use them in large scale experiments such as HTS. We circumvented the problem by employing TCR Tg mice; more than 2–3×10^7^ primary resting naïve T cells with single antigenic specificity can be obtained from whole body lymph nodes of one mouse [32].

While purified recombinant ligands are routinely used in studies of receptor/ligand interactions in both protein-based and cell-based assays, the cost of ligand expression and purification is high and the condition in which receptor-ligand interactions take place is fairly non-physiological as the purified ligands are often immobilized on the surface of plastic in those assays [33], [34]. In contrast, because the ligands used in our assay are prepared as expressed in vesicluated plasma membranes, the ligand preparation cost is cheaper and receptor/ligand interactions take place in a more physiological condition.

Even though flow cytometric analysis holds advantages of superior sensitivity, statistical precision and concomitant examination of multiple parameters, it has been less popular in HTS due to its limitation in throughput of sample analysis and lack of automatic sampling devices. Those issues have been resolved considerably in recent years by the invention of affordable automatic high throughput samplers (BD Bioscience). Here, a high throughput flow cytometry system developed by Dr. Sklar and his colleagues deserves a special attention [35].

The pilot scale HTS validated the usefulness of our HTS platform in the systematic investigation of molecular mechanisms controlling TCR-mediated membrane-proximal signaling cascades leading to LFA-1 activation. Besides hit compounds mentioned in the Results section, it is also of note that several mitochondrial antagonists, i.e., a group of rotenoids typified by Rotenone and Deguelin [36] and Antimycin [37] (Figure 4), were isolated as hits with very high potencies; IC_50_ values of the rotenoids were in the range of 200–400 nM and that of Antimycin was <10 nm. While the molecular mechanism for the inhibition of the pMV-absorption by those mitochondrial antagonists is yet to be understood, it was suggested that the inhibition might be related to AMP-activated kinase [38], whose activation was ubiquitously observed following the mitochondrial antagonist treatments (author's unpublished data).

It is intriguing that structurally-related H1 histamine receptor antagonists and antidepressants promoted 2C T cell absorption of the pMVs. Here, it has to be mentioned that studies by others [39] have shown activation of specific T cell signaling events after Fluoxetin treatment; e.g., activation of protein kinase C, induction of Ca^2+^ entry. In addition, Zimelidine, the second generation serotonin-specific reuptake inhibitor (SSRI), had been withdrawn out of the market after finding that it could have caused severe side effects of hypersensitivity reactions such as Guillain-Barre syndrome, a life-threatening neurological autoimmune disease, and flu-like syndromes [29]. Even though such side effects are not widespread among other antidepressants, it has been a concern that the structurally related antidepressants may cause similar adversary effects as Zimelidine in certain conditions. Our results support the notion that the structurally-related antidepressants may modulate T cell activation process.

## Materials and Methods

### Ethics Statement

All procedures met the guidelines of the National Institutes of Health “Guide for the Care and Use of Laboratory Animals”.

### Animals

C57BL/6J (B6), CD28^−/−^ (B6) and CD18^−/−^ (LFA-1^−/−^) (B6) mice were purchased from The Jackson Laboratory and maintained at The Scripps Research Institute (TSRI). 2C TCR transgenic mice [32] were bred at TSRI. 2C TCR Tg mice deficient in CD28 (2C.CD28^−/−^) and CD18 (2C.LFA-1^−/−^) expression were generated in our laboratory.

### Cell lines and culture media

Dros APCs were maintained in Schneider's medium supplemented with 10% fetal bovine serum (FBS) plus antibiotics as described [8].

### Peptides, chemicals and mAbs

QL9 (QLSPFPFDL), p2Ca (LSPFPFDL) and P1A (LPYLGWLVF) peptides were purchased from Invitrogen.

PP2, Wortmannin, Cyclosporin A, Cytochalasin D, Nocodazole, BAPTA/AM, and Forskolin were purchased from EMD Bioscience. Pervanadate was prepared from Na-orthovanadate (Sigma) as described [40].

Diphenhydramine, Ranitidine, Thioperamide, Fluoxetine, Sertraline, Fluvoxamine, Paroxetine, Clomipramine, Imipramine, Desipramine, Doxepin, and GBR-12935 were purchased from Sigma. Brompheniramine maleate was purchased from MicroSource Inc. Zimelidine was purchased from Tocris Bioscience.

Anti-CD28 (37.51) and anti-CD11a (M17/4) were purchased from Biolegend. Rat mAb (RTK 2758) was purchased from Biolegend and used as an isotype control mAb. FITC-conjugated TCR-β (H57-597) mAb, PE-conjugated anti-B7-1 (16-10A1), anti-L^d^ (24-14-8), anti-ICAM-1 (YN1/1.7.4) and anti-B7-2 (GL-1) mAbs and Alexa 647-conjugated anti-CD8 (53-6.7) and anti-CD18 (M18/2) mAbs were purchased from Biolegend. Alexa 546-conjugated anti-B7-1 (16-10A1) mAb was prepared in our laboratory using Alexa 546 labeling kit (Invitrogen). FITC-labeled phalloidin was purchased from Sigma.

Polyclonal antibodies directed to pan-Akt, phospho-Akt (Thr^308^), PLC-γ1 and phospho-PLC-γ1 (Tyr^783^) were purchased from Cell Signaling Technology. Horseradish peroxidase-tagged goat anti-rabbit IgG Ab was purchased from Santa Cruz Biotechnology.

### Preparation of pMVs

PMVs were prepared as described by Goldberg and Kornfeld [41] with modifications. In brief, Dros APCs induced to express transfected mouse immuno-molecules were washed and resuspended in buffer A (15 mM KCl, 1.5 mM MgCl_2_, 1 mM DTT, 10 mM Hepes, pH 7.5) containing cocktail of protease inhibitors (Sigma) and lyzed with Contes homogenizer (loose-fitting pestle). The cell lysate was spun down briefly at low centrifugal force (1500× g) to remove unlyzed cells and nuclei. The supernatant was recovered and mixed with one fifth volume of buffer B (375 mM KCl, 22.5 mM MgCl_2_, 1 mM DTT, 220 mM Hepes, pH 7.5) containing protease inhibitors and subjected to ultracentrifugation (1 hr at 50,000× g at 4°C) to collect the crude membrane particles. The resulting pellet was resuspended in buffer C (40 mM Tris·Cl, 10 mM KCl, 10 mM NaCl, 0.2 mM EDTA, pH 7.2) and finely dispersed using Contes homogenizer (tight-fitting pestle). The resuspended membrane vesicles were further fractionated by sucrose gradient ultracentrifugation (step gradients of 20, 35, 40 and 50%); 200,000× g for 2 hrs at 0°C. The membrane vesicles derived from plasma membrane fraction were then taken from the interface of 20/35% sucrose layers, aliquoted and stored at −80°C. The amount of pMVs was determined by measuring the protein concentration in the sample.

### Estimation of the average number of each ligand expressed in the pMVs

The average number of each mouse ligand expressed in L^d^B7-1ICAM-1 pMVs was estimated, based on the number of each ligand expressed in the intact Dros APCs and average surface areas of the Dros APCs and the pMVs. The average number of each ligand expressed in the intact Dros APCs was first estimated using Quantum™ Simply Cellular Kit and Quickcal software (Bang Laboratories, Fisher, IN) following manufacturer's manual. The average number of each ligand expressed in the pMVs was then calculated as follows; the average number of each ligand expressed in the pMVs = (the average number of each ligand expressed in the intact Dros APCs) × (average surface area of pMVs)/(average surface area of intact Dros APCs). The average diameter of Dros APCs is about 20 µm.

### Purification of 2C TCR Tg T cells

2C T cells were negatively purified from pooled lymph nodes using CD8a^+^ T Cell Isolation MACS™ kit (Miltenyi Biotech). If needed, the MACS purified 2C T cells were further purified by panning on anti-CD8 mAb (3.168)-coated plates to isolate CD8^+^ 2C T cells.

### Assay for 2C T cell absorption of L^d^B7-1ICAM-1 pMVs

Forty five microliters of purified 2C T cells (1×10^6^/ml) in a pre-warmed culture medum (DMEM medium plus 10% FCS) were mixed with 5 µl of the peptide-loaded pMVs (0.5 mg/ml) in round-bottom 96 well plate and cultured for 30 min in a 37°C humidified CO_2_ incubator. After culture, PE-conjugated anti-B7-1 plus Alexa 647-conjugated anti-CD8 mAbs were added and the plate was incubated on ice for 20 min. The plate was then filled with ice-cold FACS buffer (1× PBS, 1% BSA, 2.5% horse serum, 2 mM EDTA) and spun down for cell washing. The T cells were then fixed with 2% PFA overnight at 4°C before analysis by flow cytometry. Alternatively, the T cells were resuspended in the FACS buffer containing propidium iodide (PI) and immediately analyzed.

For compound testing, 2C T cells were incubated with 0.5 µl of compounds in DMSO for 30 min at 37°C prior to culture with the pMVs.

### Flow cytometric analysis of F-actin contents

F-actin contents in 2C T cells were measured as described [42]. Briefly, 100 µl of purified CD8^+^ 2C T cells (2×10^6^/ml), pre-warmed at 37°C for 15 min, was cultured with 10 µl of the peptide-loaded pMVs (0.5 mg/ml). After culture, the T cells were washed with ice-cold 1× PBS and fixed with 4% paraformaldehyde (PFA) for 1 hr at rt, permeabilized with 0.1% Tritin X-100 and stained with FITC-conjugated phalloidin plus Alexa 647-conjugated anti-CD8 mAb for 20 min at rt. After thorough washing with 1× PBS, the T cells were analyzed by flow cytometry.

### Confocal microscopy [43]

Purified CD8^+^ 2C T cells (1×10^5^) were cultured with peptide-loaded pMVs on poly-L-lysine-coated coverslips. After culture, 2C T cells were fixed with 2.5% PFA for 30 min at rt, permeabilized with 0.2% Saponin for 15 min at rt, treated with blocking buffer (1× PBS, 1% bovine serum albumin, 2.5% horse serum, 0.02% Saponin) for 1 hr at rt and stained with fluorescence-labeled mAbs for 30 min at rt. After thorough washing with 1× PBS, T cells on the coverslip were mounted on a glass slide with ImmunO Immuno-Fluore Mounting Medium (MP Biomedicals). The mounted specimens were observed under an Axiovert S100 TU™ inverted microscope (Zeiss) connected to a LaserSharp™ (Bio-Rad) confocal imaging system. The collected images were analyzed using Image J software (National Institute of Health).

### Western blot analysis

Purified 2C T cells cultured with peptide-loaded pMVs were washed with ice-cold 1× PBS and lyzed in a cell lysis buffer (50 mM Tris·HCl, 150 mM NaCl, 1% Igepal CA-630, pH 7.5) containing cocktail of protease inhibitors (Sigma) followed by sonication for further homogenization. The same amounts of lysates (determined by the protein concentration) were separated by SDS/PAGE and subjected to Western blotting as described [44]. The proteins of interest were visualized by ECL reagent (Pierce).

## Supporting Information

Figure S1 (0.10 MB EPS)

Figure S2 (0.15 MB EPS)

Figure S3 (0.15 MB EPS)

Figure S4 (0.10 MB EPS)

Figure S5 (0.17 MB EPS)
